# Supplementary material for: Non-adherence to the World Health Organization’s physical activity recommendations and associated factors among healthy adults in urban centers of Southwest Ethiopia
Source: PLOS Glob Public Health. 2023 Jan 12;3(1):e0001451. doi: 10.1371/journal.pgph.0001451 (PMC10021407; doi:10.1371/journal.pgph.0001451)
Supplement: Attachment — Submitted filename: Response to Reviewers.docx [file pgph.0001451.s002.docx]

**Date 06/07/2014**

**To: PLOS Global Public Health and Reviewers**

**Issue: Rebuttal letter**

Dear editors of the PLOS Global Public Health and respected reviewers, I received your comments and editorial requirements on my manuscripts. I accepted all the comments and made changes to the manuscript as per the recommendations. Further, the specific responses to the issues raised by reviews are listed hereunder.

Regards

Sabit Zenu

Corresponding Author

1. Editorial Requirements
   1.1. Please insert an Ethics Statement at the beginning of your Methods section, under a subheading 'Ethics Statement'.

**Response**: The required section is added with necessary details

Methods, Ethics statement

1.2. Please amend your detailed Financial Disclosure statement. This is published with the article. It must therefore be completed in full sentences and contain the exact wording you wish to be published.

**Response**: The financial disclosure statement is now amended as recommended

1.3. If you have no competing interests to declare, please state "The authors have declared that no competing interests exist" in the Competing Interest Section.

**Response**: the competing interest statement is now amended as recommended

1.4. . In the online submission form, you indicated "All data for this research article is available and can be accessed from the corresponding author."

**Response**: the statement will be edited on the online submission system and the data will be uploaded as a supplementary material.

2. Response to reviewer 1
   2.1. Pag 3, line 62. Please add in all kind of individuals and quote adequate references as follow: Physical Activity for Health—An Overview and an Update of the Physical Activity Guidelines of the Italian Ministry of Health. J. Funct. Morphol. Kinesiol. 2016, 1, 269-275. <https://doi.org/10.3390/jfmk1030269>

Response: The details are amended, the recommended reference is now added to the document

3. Response to Reviewer 2
   3.1. Line 29. What type of factors did you try to identify? This information has to be clear.

**Response**: The identified factors were socio demographic factors, family related factors and social capital and cohesion related factors. The detail was left due to word restrictions in the abstract section.

3.2. Line 31. From where?

**Response**: The study was conducted among health urban residents in Southwest Ethiopia. The required information is now added to the abstract

3.3. Line 35. “In this study” can be deleted.

**Response**: The term is now deleted

3.4. Line 35. “are”, the verbs must be in the past. Revise the entire document.

**Response**: The tense problem is now corrected where necessary. I thank the reviewer for the detailed comments.

3.5. Lines 49-53. The entire document must be revised. The sentences are about the present, but the references to support the statement are quite old. The sentences and the references must be accurate.

**Response**: This specific paragraph is about the public health impacts of physical inactivity and are details of general truths which we think should be presented in a present tense.

3.6. Line 54. The first sentence needs a reference.

**Response**: Reference is now added to the statement

3.7. Lines 58-62. These sentences need the references of the WHO. Moreover, the recommendation is not accurately described. The text must be amended.

**Response**: the references are now put in the recommended places

3.8. Revise the entire introduction. References have to be put after the sentences, not just at the end of the paragraphs.

**Response**: The references are now amended in the entire introduction section

3.9. There is no information about the factors related to PA in the methods section. This information is important. The readers have to know the variables and how the data were collected. This is a big flaw.

**Response**: The variables are collected using the GPAQ, EDHS questionnaire, and Integrated Social capital measurement questionnaire validated for use in developing countries. This information is now added to the methods section.

3.10. Lines 139-141. How ethnicity and religion were asked?

**Response**: These two variables are asked as per the questions meant to measure them on the Ethiopian Demographic and Health Survey data collection tool.

3.11. Lines 147-164. It is not clear how these results appear in the article. There is no reference to these factors in the methods, and it is not clear which instruments were used to measure them.

**Response**: Details of how these variables were measured is now added to the methods section

4. **Response to Reviewer 3**
   4.1. Abstract:

- Please consider specifying the details of educational status and level of happiness to present the AOR. For example, female sex had the higher odds to be physically inactive. How about educational status and level of happiness (lower is a risk or a preventive factor)?

**Response**: The direction of the predictor variables are now added to the result section of the abstract.

4.2. Introduction:

- This section consists of 8 paragraphs. In my opinion, they are too many. Some short paragraphs present the similar point which can be combined into one paragraph.

**Response**: The number of paragraphs in the abstract section is now reduced by merging some of them as per the recommendations

4.3. In this section, there are three important terms, ‘physical activity’, ‘physical inactivity’, and ‘non-adherence to physical activity recommendations’. In this context, physical inactivity = non-adherence to physical activity recommendations. I would recommend the authors to define these terms, then, use them in a consistent manner through the entire manuscript.

**Response**: Dear reviewer, thank you for your recommendations. The authors consider ‘physical inactivity’ as a harsh statement indicating complete absence of physical movement. Rather than using the term physical inactivity, we used the term ‘non adherence to the physical activity recommendations’ since some level of physical activity would normally occur short of meeting the recommendation.

4.4. Methods:

- I would recommend the authors to break down this section into sub-sections. A reporting guideline (e.g., STORBE: https://www.equator-network.org/reporting-guidelines/strobe/) may be helpful to organise the contents.

**Response**: The method section is now divided into sections as per the recommendations

4.5. - Please consider presenting the details of the questionnaire. It seems the questionnaire included other contents beyond the GPAQ. I strongly recommend presenting the validity and reliability of the data collection tool.

**Response**: In addition to the GPAQ, EDHS questionnaire and integrated social capital measurement questionnaire were used. The EDHS tool is a nationally validated tool to assess socio demographic variables in Ethiopia. We also used the SCIQ to measure some social and social capital related factors.

4.6. Please present the major variables collected by the questionnaire and how to analyse the variables. The methods for analysing the data collected by GPAQ should be described.

**Response**: The method of analysis used to compute the physical activity status is now added to the data analysis section of the Materials and Methods

4.7. - Please consider combining Table 2 and Table 3. The contents in Table 2 are quite similar to Table 3. The percentages of non-adherent and adherent should be inserted for all variables in Table 3.

**Response**: The two tables are merged as recommended

4.8. Table 3: why are some AORs missing? For example, the COR of educational status was 1.2 (0.8-1.83) (non-significant), its AOR was calculated. However, the AOR of marital status was not present.

**Response**: Since the backward stepwise multivariable logistic regression is used, only the variables with significant association in the final model were presented with the AOR values. Variables with significant association in the bivariable model but not in the multivariable model will not appear in the final output.

4.9. Discussion:

- Line 178-183: Why did the authors choose Papua New Guinea and India as comparators? I would recommend the authors to emphasise the points of comparisons, for example, comparing between the study findings with other studies in Ethiopia and in the African region. Otherwise, comparing between urban and rural populations in other countries may be helpful to explain the differences of this current study and the previous study conducted in rural Ethiopia.

**Response**: Dear reviewer, thank you for your insight. These studies are used in discussion due to the shortage of comparable studies with methodological similarities in Ethiopia and neighbouring African countries. Despite the geographical disparities, these setups are comparable in general terms as they are all in developing countries

4.10. Please consider the limitations of this study. The present limitation is unclear. What is the main problem of GPAQ compared with an objective measurement of physical activity? Are there any other limitations?

**Response**: Additional limitations like the possibility of recall bias is added as a limitation.

4.11. References:

- Please check the consistency of the references (e.g., journal abbreviations, small and capital letters).

**Response**: The references are now edited and corrected according to the requirements of PLOS journals.
