# Supplementary material for: Non-adherence to the World Health Organization’s physical activity recommendations and associated factors among healthy adults in urban centers of Southwest Ethiopia
Source: PLOS Glob Public Health. 2023 Jan 12;3(1):e0001451. doi: 10.1371/journal.pgph.0001451 (PMC10021407; doi:10.1371/journal.pgph.0001451)
Supplement: Attachment — Submitted filename: Response Letter.docx [file pgph.0001451.s003.docx]

**Response to editorial requirements and reviewer comments**

A. **Editorial Requirements**
1. Please review your reference list to ensure that it is complete and correct. If you have cited papers that have been retracted, please include the rationale for doing so in the manuscript text, or remove these references and replace them with relevant current references. Any changes to the reference list should be mentioned in the rebuttal letter that accompanies your revised manuscript. If you need to cite a retracted article, indicate the article’s retracted status in the References list and also include a citation and full reference for the retraction notice.

**Response**: All article references are checked and are at least available on the Google scholar. No retracted article is cited. The write-up of all references is updated and now standardized according to the journal requirements.

References: Page 13-16

2. Please amend your detailed Financial Disclosure statement. This is published with the article. It must therefore be completed in full sentences and contain the exact wording you wish to be published.

**Response**: The financial disclosure statement is now corrected as recommended. The recommended statement ‘The authors received no specific funding for this work’ has been used both in the manuscript and the online submission format.

Page 11: Line 280

3. We have noticed that you have uploaded Supporting Information files, but you have not included a list of legends. Please add a full list of legends for your Supporting Information files after the references list.

**Response**: Legend is now provided to the supplementary file

B. **Response to Reviewer 3**
1. Introduction: Please consider reorganising this section. The current introduction consists of 8 paragraphs. Some paragraphs are very brief. Is it possible to combine some paragraphs together and make it more concise?

**Response**: The introduction is now reorganized and reduced to five paragraphs as per your recommendation by merging relatively brief ones.

**Introduction**: Line 68-96

2. Result: Sub-section ‘Non-adherence to the WHO recommendations of physical activity’ – The old Table 2 presenting a chi-square test has been removed. Therefore, I would like to recommend the authors to add brief explanation of the chi-square test in the text.

**Response**: The chi square findings for sex and age distribution of the outcome variable are now added to the text of the manuscript

**Result**: Line 151-153

3. Result- Page 7, line 165: please replace Table 3 with Table 2 (the old Table 3 has been removed already).

**Response**: The table naming is now replaced as recommended.

**Result**: Line 157

4. Previously, I enquired about missing AORs. The authors explained that ‘only the variables with significant association in the final model were presented with the AOR values’. . I am confused by reading the data in the current Table 2. For example, Marital status (other): COR = 0.9(.63-1.4) – no AOR, while Educational Status (Primary and Below): COR 1.2(0.8-1.83), 0.51(0.3-0.9), p = 0.03* [is this COR significant?]. The wealth index (2nd to 4th) quartiles were not significant, however, their AORs were calculated. Please clarify and organise the numbers, if appropriate.

**Response**: In the analysis, we used the bivariable and multivariable logistic regression. Variables with p-values of <0.25 on the bivariable logistic regression were taken to the multivariable logistic regression. Accordingly, all variables have a COR result for the bivariable logistic regression. From the variables which were significantly associated in the bivariable model, only few remained significant in the final step of multivariable logistic regression model. We used backward stepwise logistic regression in the multivariable model, and variables whose *p*-values are >0.1 will not be transferred to the next step. In this way, the final step of the regression will only include variables that have p-values<0.1. For this reason, the final model will not calculate the AOR for some variables as they are removed step by step from the analysis.

5. References:
   - Please check the consistency of the references (e.g., journal abbreviations, small and capital letters). For example, ref#2: Global Recommendations on Physical Activity for Health for age 18- 64 years old (using capital letters at the beginning of each word) VS. ref#3: Global action plan on physical activity 2018-2030: more active people for a healthier world (using small letters except the first word). This should be consistent throughout the reference list.

**Response**: All references are now updated and standardized according to the journal requirements. The journal abbreviations are used according to their standardized names in the indexing databases. We used Mendeley reference manager and the software automatically introduces the correct journal abbreviations as it appears in the indexing databases.

**References**: Page 13-16

6. General comments:

- Please recheck the writing style/typos (e.g., don’t, didn’t).

**Response**: Thank you very much, we reviewed the manuscript and we have corrected the use of such short forms.
